# Liquid Biopsies in Renal Cell Carcinoma—Recent Advances and Promising New Technologies for the Early Detection of Metastatic Disease

**DOI:** 10.3389/fonc.2020.582843

**Published:** 2020-10-28

**Authors:** Harini Lakshminarayanan, Dorothea Rutishauser, Peter Schraml, Holger Moch, Hella A. Bolck

**Affiliations:** Department of Pathology and Molecular Pathology, University of Zurich and University Hospital Zurich, Zurich, Switzerland

**Keywords:** liquid biopsy, prognostic markers, renal cell carcinoma (RCC), translational research, tumor biomarkers

## Abstract

Clear cell renal cell carcinoma (ccRCC) displays a highly varying clinical progression, from slow growing localized tumors to very aggressive metastatic disease (mRCC). Almost a third of all patients with ccRCC show metastatic dissemination at presentation while another third develop metastasis during the course of the disease. Survival rates of mRCC patients remain low despite the development of novel targeted treatment regimens. Biomarkers indicating disease progression could help to define its aggressive potential and thus guide patient management. However, molecular markers that can reliably assess metastatic dissemination and disease recurrence in ccRCC have not been recommended for clinical practice to date. Liquid biopsies could provide an attractive and non-invasive method to determine the risk of recurrence or metastatic dissemination during follow-up and thus assist the search for surveillance biomarkers in ccRCC tumors. A wide spectrum of circulating molecules have already shown considerable potential for ccRCC diagnosis and prognostication. In this review, we outline state of the art of the key circulating analytes such as cfDNA, cfRNA, proteins, and exosomes that may serve as biomarkers for the longitudinal monitoring of ccRCC progression to metastasis. Moreover, we address some of the prevailing limitations in the past approaches and present promising adoptable technologies that could help to pursue the implementation of liquid biopsies as a prognostic tool for mRCC.

## Introduction

Kidney cancer is the seventh most frequent cancer worldwide and is responsible for nearly 100,000 deaths each year. Clear cell renal cell carcinoma (ccRCC), the most common subtype of RCC, accounts for almost 75% of detected cases and is therefore far more frequently studied than the rarer histologies (1). One of the landmark events in its tumorigenesis is loss of the short arm of chromosome 3p on which the *VHL* tumor suppressor is encoded. This is often concurrent with a gain of chromosome 5q resulting in the generation of a small population of tumor-initiating cells (2). Consequently, the inactivation of the second copy of the VHL gene heralds the development of clinically aggressive ccRCC (2). Genetically, ccRCC is characterized by high intra-tumor heterogeneity (3, 4). Recurrent somatic mutations found in ccRCCs occur in the epigenetic regulators *PBRM1*, *SETD2* and *BAP1*, all of which are also located on chromosome 3p and are therefore prone to inactivation similar to *VHL* (5). These specific genetic changes are reflected at the RNA and protein levels, for instance, by activation of the HIF-pathway and a corresponding increase in expression of angiogenesis-related mRNA signatures and hypoxic signaling, which are direct consequences of VHL inactivation (6). Extensive metabolic reprogramming is another result of the genetic changes that occur during ccRCC initiation and progression and this is increasingly recognized to correlate with aggressive disease (7). This is exemplified by the inactivation of the pyruvate dehydrogenase complex (PDC) which in turn impairs the Krebs cycle and oxidative phosphorylation resulting in a metabolic shift toward glycolysis (8). Importantly, metabolic rewiring in ccRCC has been shown to induce HIF-signaling independent of VHL through signaling pathways that involve for example mTOR and MET. This metabolic distortion could influence epigenetic changes and chromatin dysregulation, contributing to the aggressiveness of the tumor (9).

In contrast to primary ccRCCs, which often show a high number of subclonal drivers, metastatically progressed disease sites have a more homogenous molecular landscape. They contain fewer somatic mutations indicating the excerption of only those clones that are metastatically competent. Conserved trajectories have been identified to lead to metastasis, with PBRM1 mutations often predicating dissemination (10). Other hallmark genomic alterations that lead to metastasis are the loss of chromosome 9p and 14q. Interestingly, microRNA (miRNA) signatures are also disparate between the primary and metastatic sites, with several miRNAs associating with worse patient outcomes (11). A prominent example is miR-30c, which showed decreased expression in metastatic disease corresponding to lower progression-free survival (PFS). This finding is in line with its observed function in cell adhesion and invasion (12).

**Figure 1:**
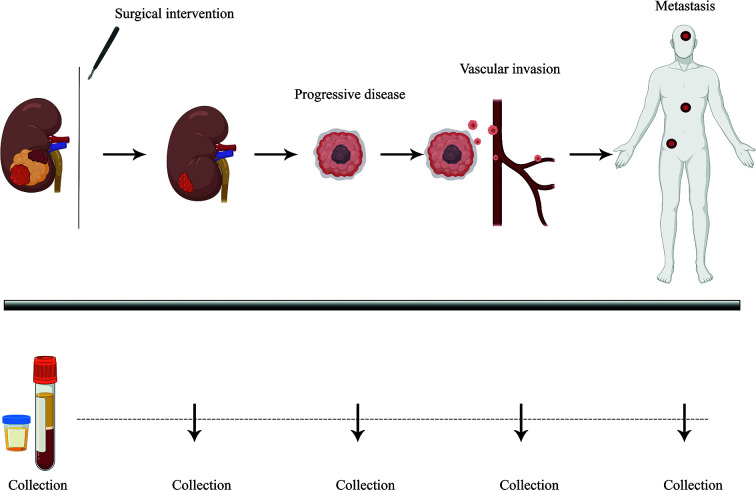
Longitudinal monitoring of disease progression *via* liquid biopsy. Liquid biopsy presents as a minimally invasive prognostic technique, allowing the surveying of disease burden and progression in patients through biological liquid samples such as blood and urine. ccRCC patients, who tend to show high variability in disease progression, could benefit from better therapeutic response and PFS (Progression-free survival) with continuous follow-up of tumor molecular profiling through analyzing tumor-specific circulating biomarkers over time. Plasma and urine samples could be collected over several time points and profiled *via* ultra-sensitive analytical techniques, helping guide clinical management strategies.

**Figure 2:**
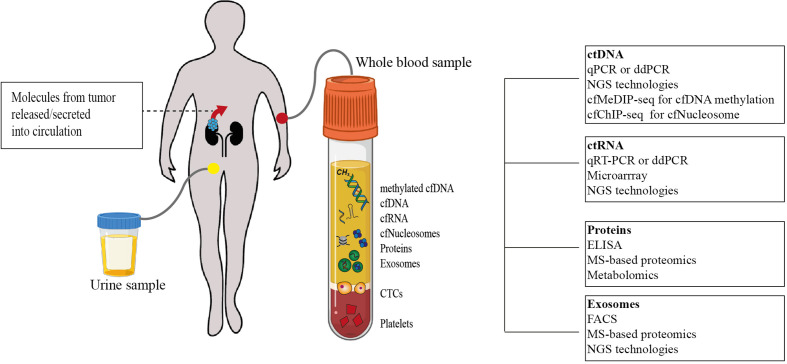
Tumor-specific circulome and technologies used for their analysis. Tumor-specific circulating biomarkers can include several molecules involved in tumorigenesis and tumor progression. Analytes that have been studied as potential biomarkers so far are depicted. Using a variety of techniques, their quantification at a single time point may allow disease staging and prognostication (cfDNA. cell-free DNA; cfRNA. cell-free RNA; cfNucleosomes. cell-free nucleosomes; CTCs, circulating tumor cells; ctDNA, circulating tumor DNA; qPCR, quantitative polymerase chain reaction; ddPCR, droplet digital polymerase chain reaction; NGS, next-generation sequencing; cfMeDIP-seq, cell-free methylated DNA immunoprecipitation sequencing; cfChIP-seq, cell-free chromatin immunoprecipitation sequencing; ctRNA, circulating tumor RNA; ELISA, enzyme-linked immunosorbent assay; MS, Mass spectrometry; FACS, fluorescence-activated cell sorting).

Importantly, the clinical diagnosis of ccRCC is most often incidental. Almost 30% of ccRCC patients already present with metastatic disease while another 30% develop metastasis later during the course of the disease (1). The prognosis for metastatic RCC (mRCC) is still relatively dismal with a variable spectrum of overall survival (OS) times ranging from less than 6 months to more than 5 years (13). It is therefore clear that accurate prognostic and risk identification strategies that enable the early prediction of recurrences could impact ccRCC clinical management (**Figure 1**). In fact, the likelihood of a favorable response to treatment is superior with limited metastatic burden (14). However, no specific molecular marker has been recommended for this clinical use to date (15). Liquid biopsies are emerging as a minimally invasive, rapid, and cost-effective tool to determine cancer markers in biological liquids such as blood or urine (**Figure 1**) (16, 17). The source for these potential biomarkers is the “circulome”, which refers to the molecules released into circulation from all tissue, including the tumor tissue. Therefore, liquid biopsies may contain tumor-specific information in the form of circulating tumor cells (CTCs), circulating tumor DNA (ctDNA), circulating tumor RNA (ctRNA), secreted proteins, extracellular vesicles, metabolites, and tumor-educated platelets. Currently, a small number of non-invasive blood tests that detect ctDNA are used as companion diagnostic tool for cancers such as non-small cell lung carcinoma (NSCLC), prostate and colorectal carcinoma. These tests are mainly used as rationales for treatment decisions, for example to detect activating mutations in the Epidermal Growth Factor Receptor (EGFR) that can be treated by administering Osimertinib in patient with NSCLC (18). Alongside, several liquid biopsy tests are under investigation in clinical trials as reviewed by Heidrich et al. (19). Among the putative markers with prognostic relevance in mRCC, ctDNA, ctRNA, proteins and exosomes are currently under heavy examination. In this review, we will provide a brief overview of the recent developments in the identification of circulating biomarkers that are indicative of a metastatic lesion and which allow the identification of disease recurrence in ccRCC patients. Moreover, we present several novel and promising technologies that could overcome some of the current limitations of liquid biopsy analysis that have been roadblocks to implementing them as a prognostic tool with clinical utility (**Figure 2**).

## Circulating Tumor DNA

The highly aggressive and vascularized nature of ccRCC prompted the intuitive expectation that tumor material, such as DNA, could be shed into circulation constituting a powerful tool to profile the tumor genome bypassing the need for a tissue biopsy. We have identified a number of studies, using the search terms “Renal cell carcinoma” and “Circulating tumor DNA” from public databases, that have investigated such possibilities (**Table 1**). Early reports paved the way by demonstrating the feasibility of genetic analysis from liquid biopsies, initially proposing the use of cell-free DNA (cfDNA) concentration and fragmentation as a guide for predicting and following the progression toward mRCC. Repeatedly, cfDNA concentrations were shown to be significantly higher in patients with advanced or metastatic disease compared to healthy individuals and patients with localized tumors (**Table 1**). Interestingly, analysis of the housekeeping gene *ACTB* as a surrogate measure of cfDNA concentration showed a consistent and significant elevation in RCC patients compared to healthy controls (21). Additionally, Wan et al. reported that the average plasma cfDNA level was significantly higher in metastatic tumors than in localized disease indicating that they could even be reflective of ccRCC progression (22). Even though these observations are noteworthy, both studies reported only moderate sensitivity and specificity for the alterations and thus further validation is required to clarify the clinical benefit of cfDNA concentrations as a circulating biomarker.

**Table 1:** Circulating Tumor DNA.

Reference	Evaluation method	No of Patient samples	Results
**Gang et al. (** 20 **)**	qPCR	Serum of 36 ccRCC patients and 42 healthy controls	Significant association between cfDNA integrity and tumor size and stage A significant difference in cfDNA fragmentation between pre and post-nephrectomy samples was observed
**Hauser et al. (** 21 **)**	qPCR	Serum of 35 RCC patients (29 ccRCC patients) and 54 healthy controls	Amplified *ACTB* ^384^ and *ACTB* ^106^ fragments were significantly higher in RCC group compared to healthy controls (ACTB-384: 1.77 vs. 0.61 ng/ml, p = 0.0003; ACTB-106:.31 ng/ml vs. 0.77 ng/ml p = 0.003). cfDNA threshold levels to distinguish between RCC patients and healthy individuals were 1.03 ng/ml for ACTB-106 (68.6% sensitivity and 70.4% specificity) and 1.70 ng/ml for ACTB-384 (57.1%, sensitivity and 81.5% specificity) The significant higher level of ACTB^384^ in RCC patients indicates that cell-free serum DNA is fragmented to a higher degree in cancer patients. Cell-free DNA levels of ACTB^384^, ACTB^106^ and DNA integrity did not correlate with clinical parameters such as tumor stage and grade
**Wan et al. (** 22 **)**	qPCR	Plasma of 92 ccRCC patients, 44 healthy controls	Decrease in cfDNA concentration in plasma samples following nephrectomy. Higher cfDNA levels in patients with metastatic disease (6.04ng/ml ± 0.72) when compared to patients with localized disease (5.29 ± 0.53, p = 0.017) or healthy controls (0.65 ± 0.29, p < 0.001) Increased cfDNA levels were associated with shorter recurrence-free survival Pre-treatment level of plasma cfDNA could predict recurrence with a sensitivity of 70.6% at specificity of 71.2%
**Bettegowda et al. (** 23 **)**	NGS	Plasma of 5 mRCC patients	<50% patients had detectable ctDNA
**Lu et al. (** 24 **)**	qPCR	Plasma of healthy individuals (n = 40), non-metastatic (n = 145), and metastatic (n = 84) ccRCC patients	The mitochondrial cfDNAs *Mito-1* and *Mito-2* were higher in metastatic than in non-metastatic patients and controls. *Mito-1* and *Mito-2* fragment concentration significantly correlated with Fuhrman grade (r_s_ = 0.209 and 0.206, p = 0.0121 and 0.014, respectively) *APP-3* fragment concentration decreased in both ccRCC groups The cfDNA integrity decreased from controls to metastatic patients.
**Corrò et al. (** 25 **)**	NGS	Plasma and serum samples of 9 ccRCC patients	It was not possible to identify genetic alterations such as the *VHL* mutation in ccRCC plasma without prior knowledge of patient-specific mutation profiles from primary tumor tissue
**Maia et al. (** 26 **)**	NGS- Gaurdant360 panel	Plasma from 34 RCC patients (26 ccRCC patients)	ctDNA was detected in 18 late-stage or mRCC patients (53%) with a median of 2 GAs per patient. *VHL* (n = 5) and *TP53* (n = 7) were the most frequent GAs. Patients with detectable ctDNA had significantly higher tumor size (8.81 vs. 4.49 cm; *P* = 0.04)
**Pal et al. (** 27 **)**	NGS - Guardant360	Plasma from 220 mRCC patients	Using an approach with great sensitivity to mutant cfDNA fragments at below 1%, GAs were detected in 79% patients. Most frequent GAs were TP53 (35%), VHL (23%), EGFR (17%), NF1 (16%), and ARID1A (12%). Mutations from non-RCC related somatic expansions like CHIP were not excluded 55% of variants were of unknown significance 45% of SNVs and indels were characterized with known significance. Distribution of GAs amongst patients were as follows: TP53- 30% VHL-32%, NF1-22%, EGFR-13%, and ARID1A-18%
**Yamamoto et al. (** 28 **)**	qPCR	Plasma from 92 ccRCC patients and 41 healthy controls	cfDNA concentration significantly higher in ccRCC group vs. healthy control (3803 vs. 2242 copies/ml, p < 0.001) and increased with TNM staging. Median cfDNA fragment size in ccRCC group significantly shorter vs. healthy control and negatively associated with PFS cfDNA showed 63% sensitivity and 78.1% specificity as diagnostic marker in ROC curve analysis
**Smith et al. (** 29 **)**	Whole genome/exome sequencing	MonRec study (43 metastatic RCC patients treated with multiple systemic therapies and longitudinal follow-up) and 90 patients from DIAMOND study (samples taken either prior to surgery or during progressive disease)	RCC is a ctDNA low malignancy, detection rates of ctDNA in patient plasma are ~30% using an untargeted sequencing strategy A sensitive personalized approach which is based on prior knowledge of individual tumor-specific mutations from matched tumor tissue could detect plasma ctDNA in ~50% of patients, ctDNA detection in plasma was more frequent amongst patients with larger tumors and in those patients with venous tumor thrombus
**Yamamoto et al. (** 30 **)**	NGS - RCC-specific gene panel (48 genes)	Plasma of 53 ccRCC patients	Targeted sequencing was carried out using plasma cfDNA and ctDNA In 30% patients, somatic mutations were detected in cfDNA. Most frequently detected mutations included TP53 (n = 6), BAP1 (n = 5), VHL (n = 5), TSC1 (n = 4), and SETD2 (n = 3. ccRCC patients with detectable ctDNA showed shorter fragment sizes of cfDNA. cfDNA fragments with *SETD2*, *BAP1*, and *NF2* mutations were significantly shorter than wild type cfDNA fragments. Detectable ctDNA and cfDNA size associated with poor PFS and CSS (long vs. short, P = .004, P = .011 and high vs. low, P = .317, P = .127, respectively)
**Bacon et al. (** 31 **)**	NGS - Roche SeqCap EZ Human Oncology Panel	Plasma from 55 mRCC patients	33.3% of patients showed evidence for the presence of ctDNA, exhibiting a somatic mutation in ≥1 established RCC gene. The estimated ctDNA fraction was 3.9% and median VAF = 3.6%. Most commonly mutated genes include *VHL* (41%), *BAP1* (29%), and *PBRM1* (17%). Mutation profiles were highly concordant between ctDNA and corresponding tissue (77% of mutations were shared) 11 patients were identified harbouring non-RCC specific cfDNA somatic mutations and lower VAF = 1.5%, arising from CHIP 22 CHIP-related mutations in all patient samples, median VAF = 2.15%. ctDNA positive patients had lower PFS and OS Evidence of somatic expansions unrelated to RCC, such as CHIP were detected in 43% of patients.

List of original articles cited in this section, with the main results summarized. The classification in RCC subtypes was not unequivocally done in all studies. Since ccRCC accounts for the majority of RCC cases, reports that did not state specifically which histological subtype was analyzed were also included. BRT, Benign renal tumors; cfDNA, Cell-free DNA; CHIP, clonal hematopoiesis of intermediate potential; ctDNA, Circulating tumor DNA; CSS, Cause-specific survival; GA, Genomic alterations; NGS, Next-generation sequencing; qPCR, Quantitative real-time polymerase chain reaction; PFS, Progression-free survival; VAF, Variant allele frequency; OS, Overall survival.

Fragmentation of cfDNA has also been studied as a diagnostic and prognostic marker in RCC patients (28, 30). Several groups performed these analyses using marker DNA fragments from genes like *ACTB*, *GAPDH* and *APP* as well as Alu short interspersed nucleotide elements and the mitochondrial DNA fragments Mito-1 and Mito-2 (20, 21, 24). Lu et al. could correlate shorter cfDNA fragments of the gene amyloid beta (A4) precursor protein (*APP*) with prognostic factors for recurrence-free and OS in patients with ccRCC and the cfDNA integrity index calculated based on the ratio of these fragment concentrations showed a decreased trend from controls to mRCC patients (24). Similarly, mitochondrial and Alu elements showed increased fragmentation and lower cfDNA integrity in RCC patients. However, when analyzing DNA integrity using *ACTB* and *GAPDH* as markers, cfDNA fragmentation was increased in RCC compared to controls (20, 21). While it has been shown that cfDNA fragmentation could be a valuable biomarker, further work needs to clarify which genetic elements have to be selected to ensure a complete visualization of the cfDNA fragment landscape and its diagnostic and prognostic potential (24). ctDNA has been identified in renal cancer patients of all stages but the probability of detection increased with the tumor size indicating that advanced disease stages may be better reflected in liquid biopsies (29). However, a number of studies reported ctDNA to be much less abundant in liquid biopsy samples from RCC patients compared with those from other cancers and several groups showed that ccRCC-specific ctDNA could be detected in only about 30%–50% patients (23, 25, 26, 29, 31). In a recent study, Bacon et al. used the Roche SeqCap EZ Human Oncology Panel to analyze the coding regions of 981 cancer-related genes in plasma cfDNA of 55 mRCC patients (31). Even this comprehensive analysis could only detect evidence for RCC-derived ctDNA, such as a somatic mutation in more than one established RCC genes, in a third of the patients. It is noteworthy that this was the first study that accounted for non-RCC specific somatic clones in liquid biopsies which can stem from clonal hematopoiesis of indeterminate potential (CHIP) by analysis of patient-matched leukocyte DNA. This could have decreased the sensitivity toward cfDNA mutations while increasing the sensitivity to RCC-derived ctDNA mutations. In addition, this study reveals that the blood-borne ctDNA fraction was as low as 3.9%, which is considerably less than in metastatic breast or lung cancer (32, 33).

Two other recent studies have reported higher numbers of ctDNA-positive patients. One of the largest studies by Pal et al., which included 220 mRCC patients, promisingly reported RCC-specific alterations in genes such as *VHL*, *TP53*, *EGFR*, *NF1*, and *ARID1A* in almost 80% of the patients when using a driver gene deep sequencing approach, with great sensitivity to mutant cfDNA fragments (<1%) (27). This apparent discrepancy in detecting tumor-specific mutations in the aforementioned study could be due to two factors: Although Pal et al. analyzed a larger cohort, only 56% of the samples were histologically characterized, of which 70% were classified as ccRCC. In comparison, the cohort used by Bacon et al. was completely histologically classified and contained 85% ccRCC patients. Moreover, Pal et al. did not account for non-RCC specific somatic clones which may be a rather large contributor to the genetic alterations observed in RCC liquid biopsies.

A second recent and sophisticated pipeline to detect cell-free tumor DNA in RCC patients’ plasma and urine samples was introduced by Smith et al. and was termed as a “personalized method” of ctDNA sequencing. Similar to other studies, sequencing of plasma cfDNA alone could identify ctDNA in a third of the RCC patients, including those with mRCC. However, when the personalized method that was based on prior sequencing of the primary tumor tissue and subsequent assessment of the known mutations in corresponding liquid biopsies was applied ctDNA detection rates improved to ~50% (29). In addition, in a small cohort of patients, the authors could show the prognostic value of ctDNA analysis by revealing that longitudinal plasma sampling could track disease progression. Another important finding from this study is that plasma ctDNA represented 90% of the disparate mutations found in multiple biopsy regions from individual tumors of two well characterized ccRCC patients and thus indicated that ctDNA could be used to circumvent tumor sampling bias that might be present in conventional tissue biopsies. This is an attractive approach to overcome the well-established tumor heterogeneity present in ccRCCs (34).

Taken together, a number of studies indicate RCC as a ctDNA-low malignancy by showing that only 30%–50% of patients benefitted from characterization of ccRCC-specific ctDNA using the currently available profiling technologies (23, 25, 26, 29, 31). Despite these drawbacks, ctDNA was detected more frequently in plasma amongst patients with larger tumors and hence longitudinal sampling could be used to monitor the course of the disease at least in a subset of advanced patients. Even though cfDNA analysis does not seem to enable straightforward surveillance at the moment, novel technologies could significantly improve this situation in the future.

## Proteins and Oncometabolites

Liquid biopsies could help to further investigate the proteomic landscape reflecting the changes triggered, for example, by extravasation of the ccRCC into circulation. Blood, abundant with proteins, is an inviting medium for exploring disease-related markers but it is technically challenging to mine tumor-specific signatures amidst highly abundant plasma proteins and other soluble factors. Studies identified with a keyword search for “renal cell carcinoma”, “liquid biopsy or plasma”, “protein or proteome” were further selected based on their diagnostic and prognostic value and are discussed in this section. A number of these studied utilized different experimental approaches in order to identify proteins with differential abundance in RCC plasma or serum relative to controls (**Table 2**; Please refer to Clark and Zhang Clin Proteom 2020 (52) for a comprehensive review). Historically, one of the most extensively studied proteins in this context is the Kidney Injury Molecule 1 (KIM1). KIM1 levels were found to be significantly increased in RCC patients (36). In fact, high grade ccRCCs showed an almost 7-fold increase in KIM1 abundance and mRCC patients displayed particularly high KIM1 levels in their plasma (47, 49). Despite the rather widespread expression of KIM1 in several renal diseases (53), circulating KIM1 showed 83% specificity in detecting early stage tumors with an increase to 97% specificity in later stages (47). While this makes it a promising biomarker, further studies need to validate the clinical utility of KIM1 as a ccRCC-specific circulating protein. Acknowledging the involvement of *VHL* mutations in ccRCC tumorigenesis, proteins downstream of the hypoxia-pathway represent a class of interesting soluble markers. For instance HIG2, a hypoxia inducible protein, was elevated approximately 3-fold in the plasma of RCC patients in an ELISA-based study and its abundance decreased drastically after nephrectomy (38). CAIX, one of the most prominent targets of the VHL-HIF-pathway, also emerged as a potential biomarker showing increased protein concentrations and activity in the plasma of ccRCC patients compared controls (45). Similarly, high IMP3 levels have been observed in RCC patients and correlated with the development of distant metastasis (48). Additionally, high levels of soluble CD27 were detected in sera of ccRCC patients and *in vitro* analyses suggested that this was triggered by the high expression of the HIF-target gene CD70 (43). These examples illustrate how the knowledge of ccRCC-specific cellular aberrations can be leveraged in the search for candidate biomarkers. Nevertheless, looking beyond the VHL-HIF-pathway, the TNF-related apoptosis-inducing ligand (TRAIL) was identified as a potential biomarker showing a 2-fold decrease in RCC patient sera and being highly predictive of venous invasion and metastasis (41).

**Table 2:** Proteins and oncometabolites.

Reference	Evaluation method	No of Patient samples	Results
**Won et al. (** 35 **)**	SELDI-TOF MS/MS	Serum of 15 RCC patients, 15 patients with other urological malignancies and 6 healthy controls	119 mass peaks were identified from all samples. Bioinformatics analysis using a predictive classifier (decision tree) was constructed with 5 distinct masses (3,900, 4,107, 4,153, 5,352, and 5,987 kDa) Decision tree correctly predicted the diagnosis of 85.7% of test samples (Sensitivity = 87%, specificity = 85%)
**Han et al. (** 36 **)**	Western blot and ELISA	Urine of 42 RCC patients	KIM1 was elevated in RCC urine samples Upon examining association between KIM1 levels and RCC, Urinary KIM1 of concentrations higher than 0.1 ng/ml was associated with a >36-fold risk of RCC, 82% sensitivity, and 90% specificity Urinary KIM1 levels decrease after surgical removal of the tumor
**Hara Tomohiko et al. (** 37 **)**	SELDI-TOF MS/MS	Serum from 40 RCC samples, 44 healthy controls and 5 patients with pyelonephritis	Significantly prominent mass peaks of 4,151 and 8,968 m/z were found in RCC samples Simultaneous recognition of both peaks discriminated RCC samples from controls at 89.5% sensitivity and 80% specificity Stage I RCC could be discrimination from healthy or later stage at 88.9% sensitivity using both mass peaks.
**Togashi et al. (** 38 **)**	ELISA	Plasma of 32 RCC patients, 20 healthy controls and 10 chronic glomerulonephritis patients	Higher plasma HIG2 in RCC (~2.5-fold increase) Decreased HIG2 post-surgery in stage I and stage II
**Xu et al. (** 39 **)**	2D gel electrophoresis, MALDI-TOF MS/MS	Serum of 20 RCC patients and 20 healthy controls	Analysis of serum from diseased and healthy patients identified 19 differentially expressed proteins Finally, 6 proteins were identified with a significant Mascot score (>66): factor XIII B, complement C3, complement C3 precursor, hemopexin, and alpha-1-B-glycoprotein.
**Yang et al. (** 40 **)**	ELISA	Plasma samples from 68 RCC patients and 39 healthy controls	Plasma VEGF levels were significantly higher in RCC patients. VEGF levels associated with lymph node invasion and/or metastases
**Toiyama et al. (** 41 **)**	ELISA	Serum of 84 RCC patients and 52 healthy controls	TRAIL levels were lower in RCC patients (55.9 vs. 103.1 pg/ml; P = 0.019) Decreased TRAIL expression associated with lymph node metastasis, distant metastasis and venous invasion
**White et al. (** 42 **)**	LC-MS/MS, western blotting	Serum of 54 RCC patients and 36 normal individuals; urine of 21 RCC patients and 9 normal individuals	Using proteomic analysis, 55 proteins were identified to be significantly dysregulated in ccRCC compared to normal kidney tissue Heat shock protein beta-1 (HSPB1/Hsp27) was confirmed in two independent sets of patients by western blot and immunohistochemistry Hsp27 was elevated in the urine and serum from RCC patients Higher tumor grades (grade III-IV) were associated with higher Hsp27 expression in patient serum (p = 0.013)
**Ruf et al. (** 43 **)**	ELISA	Serum of 54 ccRCC patients and 17 healthy controls	High levels of soluble CD27 in patients with CD70-expressing ccRCC cells and CD27^+^ Tumor infiltrating lymphocytes CD70 expression levels in tissue were not reflected in sera (n = 31)
**Zhang et al. (** 44 **)**	Western blot, ELISA and iTRAQ-labelled MS/MS	Serum of 40 RCC patients, 10 healthy controls and 20 patients with other urological malignancies	16 proteins increased >1.5-fold and 14 proteins decreased <0.67-fold in RCC patients compared to controls. Quantification by western blot showed that HSC71 was significantly upregulated in RCC sera (P = 0.0037) HSC71 was elevated in RCC sera when measured with ELISA (P = 0.0028 vs. control, P = 0.0008 vs. non-RCC) and showed diagnostic value (AUC = 0.86 and 87% sensitivity at 80% specificity)
**Lucarini et al. (** 45 **)**	Western blot, ELISA and enzyme activity assay	Plasma of 8 ccRCC patients, 8 BRT and 8 controls	Plasma CAIX levels were significantly higher in ccRCC patients (p ≤ 0.005) CA IX activity was lower in healthy controls compared to ccRCC or BRT (kcat 5.57 × 10^4^ s^−1^ vs. kcat 1.62 × 10^6^ s^−1^ or 1.46 × 10^4^ s^−1^)
**Knott et al. (** 46 **)**	UPLC-MS/MS	Serum samples from 5 ccRCC patients and 5 healthy controls	Renal carcinoma cell lines were used to define a panel of 21 tumor-specific metabolic features and these were assessed in human serum samples. 9 of these features were present in serum samples. A PCA model based on these 9 feature panel provided showed diagnostic value, utilizing 2PCs at a total variance of 70.87%
**Kushlinskii et al. (** 47 **)**	ELISA	Plasma of 99 ccRCC patients, 14 BRT and 29 healthy controls	KIM-1 levels are elevated in ccRCC patients and BRT KIM-1 levels could discriminate ccRCC at all stages: Stage I: 81% sensitivity; Stage II-IV: 97% sensitivity KIM-1 levels correlated with tumor stage (stage 1/2 vs. stage 3/4)
**Tschirdewahn et al. (** 48 **)**	ELSIA	Plasma of 98 RCC patients and 20 healthy controls	Plasma IMP3 was elevated in RCC samples (20 ng/ml vs. 10 ng/ml median, p = 0.015) IMP3 levels were higher in plasma from metastatic patients High IMP3 plasma levels were associated with OS and CSS
**Scelo et al. (** 49 **)**	ELISA	Plasma from 190 RCC patients and 190 healthy controls	KIM-1 detected in 93% RCC samples and 70% controls Incident rate ratio for doubling of KIM-1 levels was 1.71 5-year risk of RCC increased with increased KIM-1 levels (low vs. high: 0.2% vs. 1.0%)
**Wang et al. (** 50 **)**	Multiplex Luminex assay	Plasma samples from 182 ccRCC patients	High levels of soluble LAG3 were associated with an increased risk of advanced disease (OR = 3.36, P = 0.002) High soluble PD-L2 concentration correlated with an increased risk of disease recurrence (HR = 2.51, P = 9.33 × 10^−4^) Patients with high soluble BTLA and high soluble TIM3 showed an increased risk of tumor-related death (6-fold increase) and decreased OS (log-rank P = 9.81 × 10^−8^ and log-rank P = 6.29 × 10^−5^, respectively)
**Zhang et al. (** 51 **)**	LC-M/MS	Urine samples from 39 RCC patients, 22 BRTs and 68 healthy controls	79 metabolites with differential abundance were identified. Pathway analysis showed disturbance in amino acid metabolism, including phenylalanine metabolism, lysine degradation, lysine biosynthesis and histidine metabolism in renal tumors 16 metabolites showed good diagnostic clinical value. Cortolone, testosterone and l-2-aminoadipate adenylate levels could distinguish malignant from benign tumors. A logistic regression model based on this panel of metabolites could discriminate RCC patients from controls with a specificity of 100% and a sensitivity 75% in the test cohort (n = 68). In an independent validation cohort, both sensitivity and specificity were 80% (n = 49) 56 metabolites were differentially expressed between RCC and normal in this validation cohort. Finally, a panel with aminoadipic acid, 2-(formamido)-N1-(5-phospho-d-ribosyl) acetamidine and alpha-N-phenylacetyl-l-glutamine could predict RCC specificity of 75% at 93% sensitivity (AUC = 0.885)

List of original articles cited in this section, with the main results summarized. The classification in RCC subtypes was not unequivocally done in all studies. Since ccRCC accounts for the majority of RCC cases, reports that did not state specifically which histological subtype was analyzed were also included. AUC, Area under curve; BRT, Benign renal tumors; CSS, Cause-specific survival; ELISA, Enzyme linked immunosorbent assay; iTRAQ, Isobaric tag for relative and absolute quantitation; HR, Hazards ratio; LC, Liquid chromatography; MALDI, Matrix-assisted laser desorption/ionization; MS, Mass spectrometry; OR, Odds ratios; OS, Overall survival; PCA, Principal component analysis; TOF, Time of flight; SELDI, Surface-enhanced laser desorption/ionization; UPLC, Ultra-performance liquid chromatography.

Despite their initial promise, none of these circulating protein markers were clinically approved. Subsequently, large-scale proteomic technologies were utilized in order to provide a deeper characterization of ccRCC-specific protein assisting the search for candidate liquid biomarkers (**Figure 2**). An earlier study could distinguish RCC patients from non-RCC and healthy controls by using SELDI-TOF and applying pattern analysis based on five proteins with masses in the range of 3,900–5,900 Da (35). Similar studies have identified other peaks at 4,151 and 8,968 m/z that significantly differed between RCC and healthy controls and had an overall specificity of 80% (37). These even provided evidence for the utility of individual proteins including factor XIIIB, complement C3, misato homolog 1, hemopexin, alpha-1-B-glycoprotein (39) and HSC71 (44) as RCC-specific soluble biomarkers. Moreover, using MALDI-TOF, RNA-binding protein 6 (RBP6), tubulin beta chain (TUBB), and zinc finger protein 3 (ZFP3) were found to reduce following surgical intervention (40). Taken together, these findings underscore the opportunity to use the plasma proteome for longitudinal disease monitoring.

Much like it has been utilized with ctDNA, linking liquid biopsy protein profiles to those of the primary RCC could provide important complimentary data for the search of candidate biomarkers. In an interesting discovery study, White et al. used LC-MS/MS analysis to identify differentially expressed proteins in ccRCC compared to normal kidney tissue (42). From this analysis, heat shock protein beta-1 (HSPB1/Hsp27) emerged as a promising candidate and consequently the utility of Hsp27 as a useful non-invasive marker was confirmed in patient sera. Besides being elevated in serum and urine of ccRCC patients, Hsp27 was also associated with high grade (Grade 3–4) tumors.

Finally, it makes intuitive sense that the assessment of soluble immune-checkpoint proteins could have the added benefit of predicting immunotherapy responses besides their diagnostic or prognostic potential alone. Soluble factors such as sLAG3, sPD-L2, sBTLA, and sTIM3 were observed in higher concentrations in ccRCC patients and were significantly correlated with survival, death-risk and recurrence (50). Moreover, these proteins have already been developed as biomarkers for immune therapy prediction in several other cancer types (54). Apart from the proteome, other oncometabolites originating from metabolic processes such as amino acid metabolism, hormone synthesis and lipid transport including leucine, N-lactoyl-leucine, N-acetly-phenylalanine, hydroxylprolyl-valine, cortolone, and testosterone have been nominated as potential liquid biomarkers in RCC patients (46, 51). A panel consisting of the metabolites cortolone, testosterone and l-2-aminoadipate adenylate was able to distinguish RCC patients from benign renal tumors with 100% specificity at 75% sensitivity, indicating an increased effectiveness in discrimination when combining groups of biomarkers that are involved in disturbed metabolic pathways (51).

Taken together, several studies have already investigated RCC-specific metabolites and in particular proteins but a large proportion of these have only examined patient samples in comparison to healthy controls in order to delineate aberrant expression patterns specific for RCC diagnosis. Even though interesting markers have been nominated, information on the prognostic value of many of these candidates is still lacking. In addition, large-scale deep proteomic characterization has revealed various potential biomarkers but due to the lack of validation in clinical cohorts, very little can be used conclusively to define a panel of markers for ccRCC monitoring. Novel mass spectrometry and multi-marker based approaches are awaited to provide more comprehensive insights and a deeper understanding of potential secretory protein markers and their prognostic value in ccRCC.

## Circulating RNA and Exosomes

Cell-free RNA either enters the blood through active release from cells in extracellular vesicles like exosomes or conjugated to proteins (55–57). Coding RNA such as messenger RNA (mRNA) as well as small non-coding RNAs like miRNA and lncRNA have presented themselves as potential liquid biopsy biomarkers (**Figure 2**). Search terms “renal cell carcinoma”, “Circulating RNA or mRNA or miRNA or lncRNA” yielded studies that were pruned to select a smaller collection with relevant clinical value for ccRCC prognosis. So far, miRNAs remain the most frequently studied class of RNA molecules probably owing to the shorter half-life of mRNA and the relative novelty of lncRNA (**Table 3**). Several miRNAs which have previously been studied in the context of cancer progression and development, have also been proposed as liquid biomarkers in ccRCC. One of the most interesting examples is *miR-210*, which is known to be regulated by the VHL/HIF-pathway (58) and has emerged as a novel indicator for ccRCC tumor burden. Elevated levels of circulating *miR-210* have been reported in patient sera and following nephrectomy they were observed to decrease in the urine of disease-free patients during follow-up (61, 62). Several other miRNAs still remain to be explored as biomarkers in circulation. Studies in primary ccRCC tissue have identified 65 miRNAs, including *miR-215*, which were significantly different between patients with mRCC and localized ccRCCs (72). Whether these miRNAs can indicate metastatic dissemination of the primary tumor in liquid biopsies has not been not investigated to date. Circulating miRNAs that have already been implicated in predicting mRCC include *miR-122-5p*, *miR-206* (63), and *miR-221*. Importantly, out of these *miR-221* has also been shown to significantly correlate with lower survival (59). In addition, the combination of serum *miR-508-3p* and *miR-885-5p* could differentiate ccRCC patients, and these miRNAs have been implicated in the positive regulation of metabolic processes such as inositol phosphate metabolism and in the Hippo and Wnt signaling pathways that have been implicated in ccRCC tumorigenesis (64). Studying these miRNAs in metastatic patients and establishing their regulatory roles in ccRCC would likely improve their value as a circulating biomarker.

**Table 3:** Circulating RNA and exosomes.

Reference	Evaluation methods	No of Patient samples	Results
**Zhao et al. (** 58 **)**	qPCR	Serum of 68 ccRCC patients and 42 healthy controls	miR-210 showed high expression in ccRCC serum and could differentiate ccRCC patients from healthy controls; 81% sensitivity, and 79.4% specificity miR-210 levels correlated with ccRCC stage and were reduced after nephrectomy.
**Teixeira et al. (** 59 **)**	qPCR	Plasma of 77 RCC patients	miR-221 and miR-222 were more abundant in RCC plasma (2^−ΔΔCt^ = 2.8, P = 0.028; 2^−ΔΔCt^ = 2.2, P = 0.044, respectively). miR-221 levels were higher in plasma of metastatic patients than patients with no metastasis (2^−ΔΔCt^=10.9, P = 0.001) and high expression correlated with lower OS (48 vs. 116 months, respectively; P = 0.024)
**Wu et al. (** 60 **)**	qPCR	Serum of 71 ccRCC patients, 8 BRT, 62 healthy controls	lncRNAs showed differential abundance: 13 lncRNAs were down-regulated and 1 lncRNAs was up-regulated in ccRCC serum. The signature of lncRNA-LET, PVT1, PANDAR, PTENP1 and linc00963 was highly specific and sensitive in discriminating between ccRCC and controls This 5-lncRNA signature was also correlated with all pathological stages of ccRCC (AUC = 0.85 and 0.8 for stage I and II-IV, respectively)
**Li et al. (** 61 **)**	qPCR	Urine of 75 ccRCC and 45 healthy controls	Urinary miR-210 was significantly elevated in ccRCC samples and discriminated ccRCC from healthy controls, 57.8% sensitivity, and 80% specificity. miR-210 levels decreased after surgical removal of the tumor
**Petrozza et al. (** 62 **)**	qPCR	Urine of 38 ccRCC patients	miR-210 was upregulated in ccRCC samples and levels significantly decreased after nephrectomy
**Heinemann et al. (** 63 **)**	NGS- Small RNA sequencing	Serum of 86 ccRCC, 55 BRT, 28 controls	2588 miRNAs were detected from which 29 miRNAs were differentially expressed between healthy and disease samples: 17 miRNAs were up-regulated and 12 miRNAs were down-regulated in the tumor samples. Serum miR-122-5p and miR-206 (log2 fold change − 1.55; p = 0.002 and log2 fold change − 1.56; p < 0.001, respectively) were down-regulated in ccRCC sera. miR-122-5p and miR-206 could discriminate ccRCC from controls miR-122-5p significantly increased in mRCC Elevated serum miR-122-5p and miR-206 correlated with shorter PFS, CSS and OS
**Liu et al. (** 64 **)**	qPCR	Serum of 10 ccRCC patients, 10 healthy controls	miR− 141−3p and miR− 508−3p were down-regulated while miR− 885−5p and miR− 592 were up-regulated in ccRCC samples. All 4 miRNAs could discriminate RCC samples from healthy donors (AUC = 0.73, 0.86, 0.91, and 0.78, respectively) The combinations of miR− 508−3p and miR− 885−5p analysis improved the discriminative power between healthy and diseased samples (AUC = 0.9)
**Simonovic et al. (** 65 **)**	qPCR	Plasma from 10 mRCC and 6 ccRCC patients, 7 healthy controls.	CDK18 and CCND1 mRNAs were less abundant in the plasma of ccRCC patients (2.1 fold change, p = 0.001 and 1.55 fold change, p = 0.039, respectively)
**Exosomes**			
**Raimondo et al. (** 66 **)**	LC-MS/MS	Urine of 29 RCC patients and 23 healthy controls	Proteomic analysis was performed on 9 urinary exosome pooled samples and led to the identification of 261 proteins from control samples and 186 from RCC patient samples. Most of the identified proteins are membrane associated or cytoplasmic A panel of 10 proteins (CD10, CP, DPEP1, MMP9, EMMPRIN, CAIX, Syntenin 1, PODXL, AQP1, DKK4) that were differently abundant in tumor and normal EVs were validated by immunoblotting
**Butz et al. (** 67 **)**	qPCR	109 ccRCC patients, 24 BRT and 33 healthy controls	The combination of miR-126-3p and miR-449a or miR-24b-5p could distinguish ccRCC from controls
**Qu et al. (** 68 **)**	qPCR	Plasma of 71 RCC patients	The non-coding transcript lncASR was increased in RCC patients lncASR levels decreased after nephrectomy and increased again upon relapse
**Du et al. (** 69 **)**	qPCR	109 RCC patients	miR-190b, miR 26a-1-3p, miR-let-7i-5p, miR-145-3p, miR-200-3p, and miR-9-5p associated with OS in an initial test cohort (n = 44) In an additional validation cohort, association with OS was verified for miR-let-7i-5p, miR-26a-1-3p, and miR-615-3p.
**Jingushi et al. (** 70 **)**	LC/MS, Western blotting	Serum of 19 ccRCC patients and 10 healthy controls	Extracellular vesicles (EVs) directly isolated from surgically resected ccRCC tissues and adjacent normal renal tissues were analyzed with quantitative LC/MS. This analysis identified 3,871 tissue‐exudative EV proteins, among which azurocidin (AZU1) was highly enriched in tumor EVs (fold‐change = 31.59). AZU1 content in EVs was significantly higher in ccRCC patients compared to those from healthy donors. Subsequent functional analyses indicated that EV‐AZU1 could be engaged with vesicle‐mediated hematogenous metastasis of RCC.
**Zhang et al. (** 71 **)**	qPCR	82 ccRCC patients, 80 healthy controls	Exosomal miR-210 and miR-1233 significantly higher in ccRCC, and higher in each stage compared to normal miR-210 and miR-1233 significantly lower post-surgery miR-1233 had higher discriminatory capability with higher specificity and sensitivity than miR-210.

List of original articles cited in this section, with the main results summarized. AUC, Area under curve; CSS, Cause-specific survival; LC, Liquid chromatography; MS, Mass spectrometry; NGS, Next-generation sequencing; OS, Overall survival; PFS, Progression-free survival; qPCR, Quantitative real-time polymerase chain reaction.

Messenger RNA is a crucial intermediate in relaying genetic changes to the protein level and may therefore reflect mutational and regulatory changes in the tumor. However, technical difficulties in detecting tumor-specific mRNA in patients’ blood has limited its development as a biomarker for disease monitoring. Recently, novel sequencing technologies have provided impetus to further investigate the potential of circulating mRNA and consequently CDK18 and CCND1 messengers were shown to be downregulated in blood of ccRCC patients (65) (**Table 2**). Similarly, an increase in lysyl oxidase (*LOX*) expression marked metastatic samples from the same cohort (65). In addition, circulating lncRNA (73), one of the newer players in the field of small non-coding RNAs, also showed promise as a RCC-specific biomarker. A signature of 5 lncRNAs (*lncRNA-LET*, *PVT1*, *PANDAR*, *PTENP1*, and *linc00963*) could distinguish RCC samples from controls with a specificity of 91% at 67% sensitivity in a training set independent of stage classification. An increase to 76% sensitivity was observed when the training set was limited to controls and stage I ccRCC patients, indicating good discrimination even for less advanced patients (60).

Exosomes are nanoscale secreted membrane-bound vesicles that play a role in cellular communication by transferring signaling molecules as packaged cargo. One of the most frequent cargo is miRNA, while several other molecules including DNA, proteins and other classes of RNAs are transported in exosomes as well (74). Studied far more in urine than in blood, these vesicles are observed to contain molecules that are capable of differentiating and identifying ccRCC and mRCC. Comparable to circulating *miR-210*, exosomal *miR-210* was elevated in ccRCC patient sera and could distinguish ccRCC patients from healthy individuals, albeit only with a specificity of 62% (71). Considering that circulating levels of miR-210 have been described as relevant biomarkers in the context of cell-free analytes and as exosomal cargo, this is nevertheless one of the more promising molecules warranting further studies to assess its diagnostic and prognostic potential as a liquid biomarker for the clinical routine. Several other exosomal miRNAs or their combinations such as enumerated in **Table 2** were also able to differentiate RCC patients from healthy controls and supported the proposed use of exosomal cargo as potential biomarkers in ccRCC (67, 69). Additionally the non-coding transcript *lncARSR* (activated in RCC with Sunitinib Resistance) that is transmitted *via* exosomes, showed increased levels in the serum of RCC patients, decreased after tumor resection and subsequently increased again during tumor relapse making it an interesting candidate for non-invasive disease monitoring (68). A small number of studies have investigated the potential of exosomal protein markers (66, 70) giving first insights into their differential abundance between healthy and tumor patients. Interestingly, comprehensive protein cargo analysis by LC/MS revealed that azurocidin (AZU1) was significantly enriched in tumor‐derived exosomes and these may even play a functional role in driving metastatic dissemination (70).

Despite the initial reports, neither lncRNA nor exosomal miRNAs or proteins have been thoroughly investigated as potential biomarkers for the metastatic disease yet. Large sample volumes and complicated and expensive processing set-ups appear as major roadblocks in this search. Therefore, technical advances are needed to improve the current approaches and pave the way to further investigate and translate RNA- or exosome-based cancer detection in the clinical setting.

## Future Avenues in Liquid Biopsy

Evidently, the field of liquid biopsy analysis is quickly evolving and has shown considerable promise for anticipating cancer progression, for example in lung, breast and colorectal cancer (75–77). However, currently there is still insufficient evidence for the clinical utility of majority of the circulating molecules in many advanced cancers as well as in ccRCC (78). Several approaches are under heavy investigation and their successful implementation could provide further rationales for using liquid biopsies as a tool for ccRCC patient management (**Figure 2**). However, one of the most important aspects for consideration in any further developments is the need to validate the emergence of potential liquid biomarkers in larger patient cohorts in order to consider them as specific and sensitive non-invasive markers of clinical utility. Currently, many interesting studies have sought to assess differential features between samples from healthy and diseased individuals. Significantly more work will be required to obtain a deeper understanding of the prognostic potential of the candidate markers as they should not only be prioritized based on their discriminatory benefit but also for their value in disease monitoring, e.g., to indicate metastatic dissemination.

One of the rather unexpected obstacles in developing a clinically applicable liquid biopsy analysis platform was the low ctDNA abundance that has so far hindered ctDNA from becoming a simple alternative to tissue biopsy in diagnosing and tracking mRCC (23, 25, 26, 29, 31, 79). Nevertheless, mutant fragments derived from RCC cells have been identified in patients’ plasma and the mutations closely mirrored the known landscape of the primary tumors (31). Several studies indicated that there are opportunities for liquid biopsies in the longitudinal follow-up of ccRCC patients provided additional improvements will be made in isolation and detection approaches. Among the main challenges is the concomitant presence of mutant fragments from non-RCC somatic clones stemming, for example, from CHIP which was identified as a main cause for the discordance between plasma and tissue RCC samples (31, 79). Incorporating appropriate controls such as white blood cell DNA could prove to be essential to eliminate variants arising from such unrelated somatic expansions. Combining personalized mutations identified from archival tumor tissue is another attractive strategy that was proposed to improve the sensitivity of ctDNA detection in mRCC (25, 29, 79). Conversely, most recently, a novel approach that is based on genome-wide mutational signal integration has challenged the paradigm of increasing the sequencing depth of a limited set of target genes for reliable ctDNA detection (80). By placing the emphasis on broadening the mutational landscape, this genome-wide single-nucleotide variant (SNV) detection platform showed evidence for allowing ultra-sensitive detection even at low-sequencing depths as well as enabling quantitative dynamic monitoring of disease burden. Thus, this approach could prove to be an attractive alternative to overcome the low ccRCC-specific ctDNA abundance and significantly increase detection sensitivity in the future.

Epigenetic regulation presents a wide avenue to explore ccRCC specific patterns, particularly since chromatin remodelers are among the most frequently altered factors. Based on the principle that tumor cells acquire aberrant DNA methylation, cell-free methylated DNA immunoprecipitation sequencing (cfMeDIP Seq) was able to markedly improve sensitivity for detecting patients with mRCC. The assessment of top differentially methylated regions of the cell-free methylome could also distinguish ccRCC and control samples (81). Since blood cfDNA is derived from fragmented chromatin, it often remains associated with histones that may contain evidence of the epigenetic landscape of the cells they originate from (82, 83). Thus, circulating cell-free nucleosomes could become interesting targets for observing mRCC-specific changes in expression programs that are often imposed by alteration in *SETD2*, *PBRM1*, or *BAP1.* Chromatin immunoprecipitation sequencing of cell-free nucleosomes (cfChIP-seq) has emerged capable of identifying cell-of-origin expression marks as well as changes in gene activity and transcription in gastrointestinal cancers (82). Extrapolating this to ccRCC could open a new window to provide detailed information about the state of the disease from liquid biopsy analysis.

Novel proteomic technologies will also bolster the development of circulating biomarkers. To aid the acquisition of ccRCC-specific peptides, pre-fractionation with the aim to either remove high-abundance proteins or enrich certain proteins could be employed. In addition, novel MS-based proteomic approaches such as Microflow LC-MS/MS (84) and Trapped Ion Mobility Spectroscopy (TIMS) (85) constitute valuable technologies for biomarker discovery. These, combined with higher throughput, will likely help to identify mRCC-specific proteins in liquid biopsies. A promising impetus for further studies in this domain was provided with the identification of several proteins differing in abundance with the infiltration of ccRCC into the renal vasculature using nano-scale liquid chromatographic tandem electrospray ionization mass spectrometry (nLC-ESI-MS/MS) for the proteomic analysis of urine and plasma (86).

Circulating RNAs (circRNA) represent a newly discovered class of small non-coding RNAs that have recently come into light as potential biomarkers for kidney diseases. Studies leveraging primary tissue collections have identified several combinations of these circRNA that are capable of identifying ccRCC (87, 88) and also correlated with tumor grade (80). Importantly, circRNAs have already been discovered as exosomal cargo in urine and plasma from patients with Idiopathic Membranous Nephropathy (89), raising the interesting possibility that these molecules could also be studied as indicators of renal cancer. Exosomes show high potential as a useful vehicle for tracking disease progression and dissemination by virtue of their aiding intercellular communication. Due to their small size and low density, recovery from plasma or urine remains the limiting step towards straightforward isolation, detection, and quantification (90). A novel chemical affinity-based capture method has been developed for extracellular vesicle isolation (EVtrap) from plasma, which showed a 7-fold increase in capture compared to ultracentrifugation and can potentially ameliorate this problem. In a promising proof-of-concept study, phosphoproteomic analysis of RCC plasma samples revealed several proteins capable of distinguishing five RCC patients from five healthy controls (91) indicating that EVtrap could be exploited to develop additional markers for disease monitoring.

It is also becoming increasingly apparent that the best definition of tumor status and prognosis may arise from the simultaneous study of various complimentary constituents of the circulome and thus, a multi-marker based approach may prove to be useful toward developing reliable biomarkers for disease surveillance (92). Harnessing the indicative potential of several of the molecules described in this review together may in fact be key to achieving prognostic utility in ccRCC liquid biopsy profiling.

## Conclusion

Liquid biopsy analysis offers a range of complementary information through the circulome and has the potential to cause a major breakthrough in clinical oncology. In contrast to conventional tissue biopsy, it may even be able to capture a larger amount of the molecular heterogeneity described for ccRCCs and inform about aggressive clones that have disseminated toward the metastatic niche. Before this potential can be realized, a number of hurdles remain but given the rapid pace of technological development there is an air of optimism regarding its utility, especially for monitoring the metastatic progression in ccRCC patients.

## Author Contributions

HL and HB contributed to the conception of the review article. HL, HB, and DR drafted the manuscript. PS and HM critically revised the manuscript. All authors contributed to the article and approved the submitted version.

## Funding

This work was supported by the Swiss National Science Foundation (SNSF grant number 310030_166391).

## Conflict of Interest

The authors declare that the research was conducted in the absence of any commercial or financial relationships that could be construed as a potential conflict of interest.
